# An Instant Donning Multi-Channel EEG Headset (with Comb-Shaped Dry Electrodes) and BCI Applications

**DOI:** 10.3390/s19071537

**Published:** 2019-03-29

**Authors:** Jeehoon Kim, Jeongsu Lee, Chungmin Han, Kwangsuk Park

**Affiliations:** 1 Interdisciplinary Program of Bioengineering, Seoul National University, Seoul 03080, Korea; jhkim119@bmsil.snu.ac.kr; 2 Mobile Communication Business, Samsung Electronics Co. Ltd.; 130 Samsung-ro, Yeongtong-gu, Suwon-si, Gyeonggi-do 16678, Korea; sekundenstopp@gmail.com; 3 Department of Biomedical Engineering, University of Texas at Austin, Austin, TX 78712, USA; cmhan616@utexas.edu; 4 Department of Biomedical Engineering, College of Medicine, Seoul National University, Seoul 03080, Korea

**Keywords:** electroencephalogram, EEG headset, EEG sensor, rapid EEG setup, brain computer interface, steady-state visual evoked potential, auditory steady state response

## Abstract

We developed a new type of electroencephalogram (EEG) headset system with comb-shaped electrodes that enables the wearer to quickly don and utilize it in daily life. Two models that can measure EEG signals using up to eight channels have been implemented. The electrodes implemented in the headsets are similar to a comb and are placed quickly by wiping the hair (as done with a comb) using the headset. To verify this headset system, donning time was measured and three brain computer interface (BCI) application experiments were conducted. Alpha rhythm-based, steady-state visual evoked potential (SSVEP)-based, and auditory steady state response (ASSR)-based BCI systems were adopted for the validation experiments. Four subjects participated and ten trials were repeated in the donning experiment. The results of the validation experiments show that reliable EEG signal measurement is possible immediately after donning the headsets without any preparation. It took approximately 10 s for healthy subjects to don the headsets, including an earclip with reference and ground electrodes. The results of alpha rhythm-based BCI showed 100% accuracy. Furthermore, the results of SSVEP-based and ASSR-based BCI experiments indicate that performance is sufficient for BCI applications; 95.7% and 76.0% accuracies were obtained, respectively. The results of BCI paradigm experiments indicate that the new headset type is feasible for various BCI applications.

## 1. Introduction

There are various applications based on electroencephalogram (EEG) signals, such as brain computer interface (BCI), brain mapping, sleep research, clinical diagnosis, and authentication [1,2,3,4,5,6]. Multichannel measurement of EEG signals is required for such applications. However, measuring EEG signals from multiple spots on the scalp can only be implemented in a hospital or laboratory environment. This is because the preparation process for EEG measurement is time-consuming and laborious, even for experts, as localization of sites for the electrical montage, attaching electrodes to the respective spots, gel injection, and impedance checking are necessary. This complex procedure required for EEG measurement is one of the major obstacles to practical application outside of hospital or laboratory environments. To overcome this drawback and simplify the procedure, various types of EEG measurement headsets or headgears have been introduced and are currently available on the market [7]. The official website of the BNCI Horizon 2020 project, a European BCI consortium, provides a list of companies involved in the BCI field and closely related sectors [8]. 

**Figure 1:**
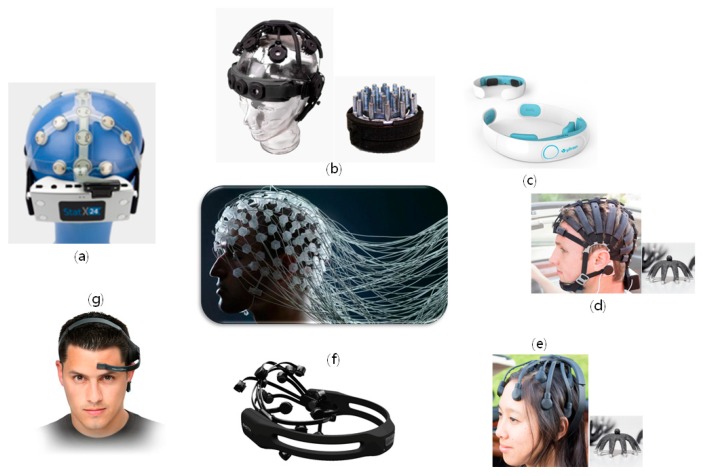
Various commercial EEG headset products on the market: (**a**) Stat X24 (Advanced Brain Monitoring), (**b**) DSI 10/20 (Quasar), (**c**) Yband (Ybrain), (**d**) HD-72 High Density Dry Headset (Cognionics), (**e**) Quick-20 Dry EEG Headset (Cognionics), (**f**) Emotiv EPOC (Emotiv), (**g**) MindWave Mobile (NeuroSky).

We investigated the companies on the list that are involved in EEG measurement headsets and found the following headsets (Figure 1). MindWave Mobile (NeuroSky Inc.; San Jose, CA, USA) and Yband (Ybrain, Seoul, Korea; not on the list) can be worn “out of the box,” but they can measure only one or two channels of EEG signals from the frontal lobe, where there is no hair. The Emotiv EPOC (Emotiv, San Francisco, CA, USA) is similar to the preceding two headsets, but conductive gel injection are necessary before use. The Stat X24 mobile EEG system (Advanced Brain Monitoring Inc.; Carlsbad, CA, USA) measures up to 24 channels of EEG signals, but it is impossible to put the headset on without assistance and gel injection is necessary in advance. The HD-72 High Density Dry Headset (Cognionics Inc.; San Diego, CA, USA) measures up to 72 channels, but assistance is required to don it. The DSI 10/20 (Quasar, San Diego, CA, USA) can be donned easily, but after donning, each electrode must be twisted for stable contact between the scalp and the electrode, which is time-consuming. The Quick-20 Dry EEG Headset (Cognionics) is quick and easy to set up, but the electrodes are spiky, which has several drawbacks of high skin-electrode impedance by small contact area as well as pain when pressing the electrodes onto the skin [9]. None of these headsets measures multichannel EEG signals immediately after donning without some level of preparation.

The functionality of EEG-based BCI is more suitable for practical, real-life applications, compared to magnetoencephalography (MEG), functional magnetic resonance imaging (fMRI), or electrocorticography (ECoG). The setup is also not as expensive and bulky as that of MEG or fMRI, and no surgery is needed, as in the case of ECoG. Hence, various paradigms for EEG-based BCIs have been developed, such as the event-related potential (ERP), steady-state visual evoked potential (SSVEP), auditory steady-state response (ASSR), slow cortical potentials, event-related desynchronization (ERD), and event-related synchronization (ERS) [10,11,12,13,14].

The mental speller is the most extensively studied BCI application. Most mental spellers for patients are implemented based on P300, which is an ERP component with an enhanced positive deflection feature and a latency of approximately 0.25 to 0.5 s, usually elicited by the “oddball” paradigm [15,16,17,18,19,20,21,22]. SSVEP, which consists of the responses of the occipital region caused by visual stimulation at specific frequencies, is also an extensively used paradigm in mental spellers [23,24,25,26,27,28,29,30]. Hwang et al. introduced an SSVEP-based BCI spelling system by adopting the QWERTY-style LED keyboard [23]. The results of an online experiment yielded an average spelling speed of 9.39 letters per minute, with an average success rate of 85.58%. 

Recently, studies on hybrid BCIs have been reported. These hybrid BCIs combine various types of existing BCIs, such as ERD and SSVEP, motor imagery and SSVEP, and EEG and non-EEG. In particular, some hybrid BCIs have combined P300 and SSVEP. For instance, Panicker et al. introduced a P300 BCI with SSVEP [31]. In their system, the SSVEP response served as a passive “brain switch.” While Panicker et al. combined P300 and SSVEP sequentially, Xu et al. and Yin et al. combined them simultaneously [32,33]. Specifically, Xu et al. proposed a 3 × 3 P300-based matrix with an SSVEP blocking feature. In their study, the SSVEP blocking feature worked as a supplementary parameter for discriminating the presence or absence of a P300 evoked potential. They reported that the combination of P300 and the SSVEP blocking feature enhanced the overall performance of the system. Yin et al. proposed a 6 × 6 P300-based matrix with SSVEP, and integrated the SSVEP into the conventional P300 paradigm. They achieved an online classification accuracy of 93.85% and an information transfer rate (ITR) of 56.44 bits/min. 

The auditory steady-state response (ASSR)-based BCI paradigm is a relatively new BCI concept, and can be classified as a vision-free BCI paradigm. In contrast to the SSVEP or the P300 paradigms, subjects do not need to move their eyes to enforce their desired commands. This could constitute a solution for patients who are completely paralyzed and cannot move their body parts, including their eyes.

In this paper, we present two multichannel EEG headsets that can be donned quickly and then measure EEG signals immediately. Our approach is focused on reducing the preparation and donning time for multichannel EEG signals measurement. The details of validation experiments conducted with the headsets are presented in the results section. Further, BCI applications are manipulated using the instant-donning headsets. Experiments using SSVEP-based and ASSR-based BCI systems were conducted with the headsets and the results compared with previous studies.

## 2. Materials and Methods

### 2.1. Electrodes

**Figure 2:**
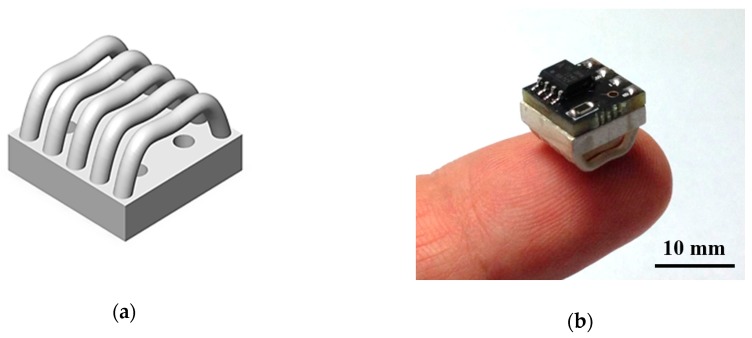
(**a**) Structure of the electrode, (**b**) photograph of electrode with preamplifier.

To develop instant donning headsets, reversed-curve arch electrodes introduced in our previous work [9] were used. The electrode was fabricated using sterling silver via a 3D printer (Perfactory, EnvisonTEC, Gladbeck, Germany). The sterling silver was an alloy containing 92.5% silver and 7.5% copper by mass. The electrode was 10 mm^2^ in size, with five reverse-curved arches. The comb-like unique structure of these electrodes increases the area of skin-electrode contact on hairy scalps and results in lower skin-electrode impedance and decreased pain [9]. Multi-channel electrodes were designed and fabricated to different heights reflecting the curvature of the head. The structure of the electrode and a photograph are presented in Figure 2a.

**Figure 3:**
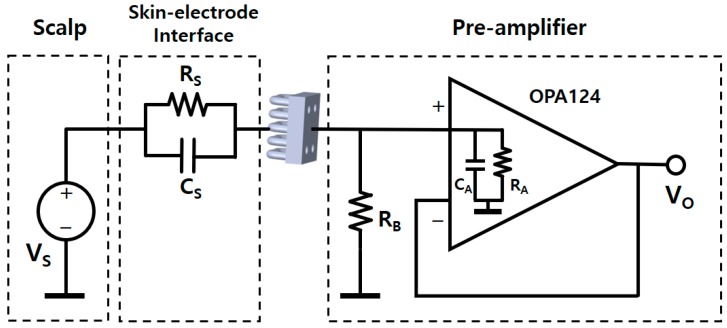
Overall equivalent circuit of the electrode and preamplifier.

A preamplifier was attached to the other side of the base of the electrode, as shown in Figure 2b. A high-input impedance op-amp (OPA124, Texas Instruments, Dallas, TX, USA), which has an input impedance of 10^13^ Ω||1 pF, was selected for use as the preamplifier. The bias resistor $R_{B}$ is used to provide a biased current path to the preamp. The value of the bias resistor is 5 GΩ. The overall equivalent electrical circuit of the electrode is shown in Figure 3. The gain of the circuit is given by

(1) $$G_{S}\left(s\right)=\frac{V_{O}}{V_{S}}=\frac{Z_{B}||Z_{A}}{Z_{S}+Z_{B}||Z_{A}},$$

where the symbol || indicates the parallel combination of two impedances, $Z_{A}$ is the input impedance of the operational amplifier ($R_{A}||C_{A}$), $Z_{B}$ is the impedance of $R_{B}$, and $Z_{S}$ is the impedance of the skin-electrode interface ($R_{S}$, $C_{S}$). $Z_{A}$ can be ignored as it is much greater than $Z_{B}$. The gain can thus be expressed as

(2) $$G_{S}\left(s\right)=\frac{Z_{B}}{Z_{S}+Z_{B}}.$$

Here, because $Z_{S}$ is the parallel combination of a resistor and a capacitor, $Z_{S}$ can be rewritten as

(3) $$Z_{S}=\frac{R_{S}}{1+sR_{S}C_{S}}.$$

Therefore, the overall source-to-output voltage gain (G(s)) can be rewritten as

(4) $$G\left(s\right)=\frac{R_{B}}{\frac{R_{S}}{1+sR_{S}C_{S}}+R_{B}}=\frac{R_{B}}{\begin{array}{c} \frac{R_{S}+R_{B}\left(1+sR_{S}C_{S}\right)}{1+sR_{S}C_{S}} \end{array}}=\frac{R_{B}\left(1+sR_{S}C_{S}\right)}{\begin{array}{c} R_{S}+R_{B}\left(1+sR_{S}C_{S}\right) \end{array}}$$

**Figure 4:**
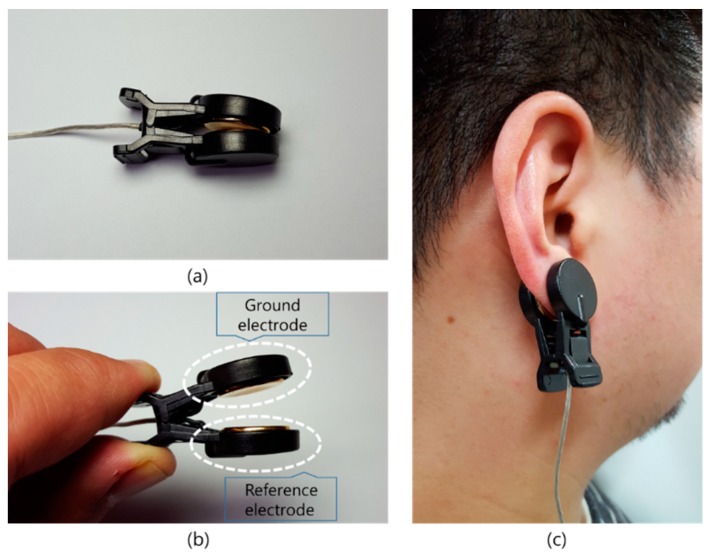
(**a**) Side view of the earclip, (**b**) ground and reference electrodes in the earclip, (**c**) photograph of the earclip worn on the earlobe.

To minimize the set-up time, ground and reference electrodes also should be considered. An earclip (LXEL-EAR-01, Laxtha, Daejeon, South Korea) (Figure 4) with ground and reference electrodes was used. The clip has a gold coated circular (diameter = 15 mm) electrode on each arm. These electrodes were originally electrically connected, but were separated from each other to make separate ground and reference electrodes.

### 2.2. Headsets

**Figure 5:**
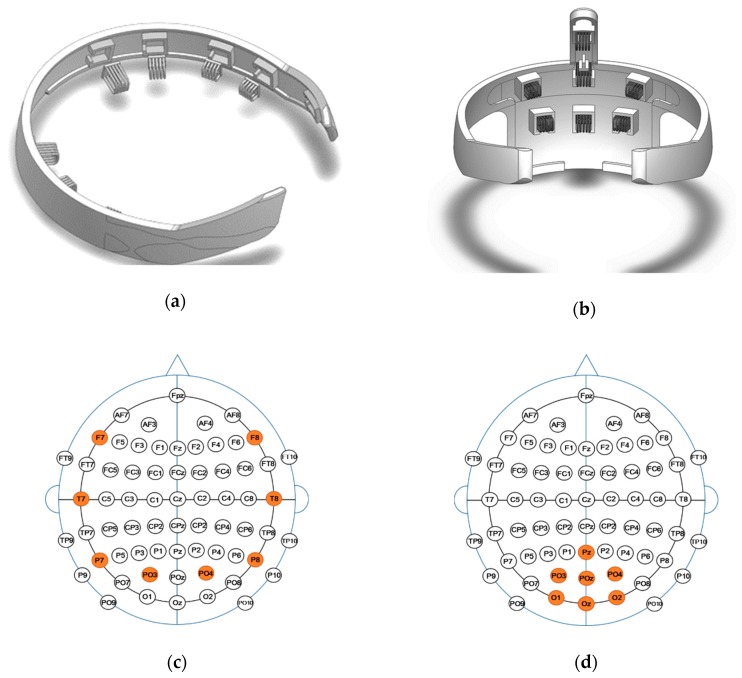
Two 3D models of the around-type EEG headset (**a**), and posterior-type EEG headset (**b**), as well as, the EEG montage of the around-type headset (**c**), and posterior-type headset (**d**). The shaded areas from the montage are the positions of electrodes used in each headset.

Two types of headsets were designed for BCI application: (1) an around-type that extends to the transversal plane of the head; (2) a posterior-type that covers the occipital and parietal region. Each headset type has two sizes, respectively, small and large for different size of the head. These headsets have been designed such that all electrodes are able to reach the scalp surface while being placed on the head; this capability eliminates the need for preparation procedures and allows EEG signal measurement immediately after donning. Two 3D models of the headsets are illustrated in Figure 5a,b. The headsets were designed in the SOLIDWORKS® environment and fabricated in plastic using a 3D printer. They have square holes for the electrodes to be affixed. All electrodes are aligned in the vertical direction of their arches (Figure 5a,b). Based on the 10-20 international system, the locations of EEG measurements with the around-type headset are “F7,” “T7,” “P7,” “PO3,” “PO4,” “P8,” “T8,” and “F8” (Figure 5c). For the posterior-type, the locations are “Pz,” “PO3,” “POz,” “PO4,” “O1,” “Oz,” and “O2” (Figure 5d). For the around-type headset, all electrodes are able to reach the surface of the scalp by being combed through the hair and toward the scalp from a point several centimeters above the crown of the head. For the posterior-type headset, by combing through the hair and toward the scalp from the crown of the head to the measurement spots, all electrodes are able to reach the surface of the scalp while the headset is being donned. By combing with the headsets, the hair smoothly goes into the gaps between the arches of the electrodes and the electrodes easily reach the surface of the scalp.

**Figure 6:**
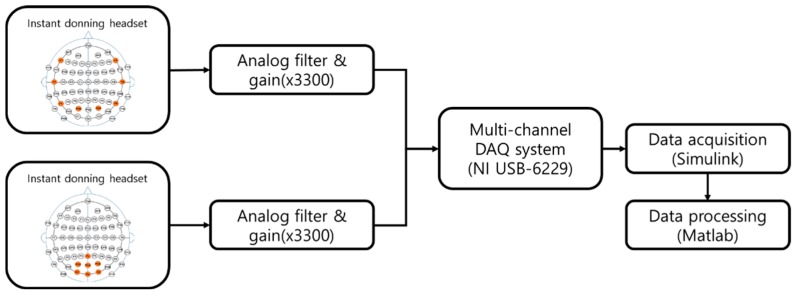
Overall schematic of the data processing system for the EEG signals from the headsets.

The signal from each electrode goes to the positive input of an instrumentation amplifier (INA118, Texas instruments Inc., Dallas, TX, USA) while the signal from the reference electrode goes to the negative input of the amplifier. The difference of these two signals is amplified with a gain of 11 in the amplifier. The output signal is sequentially filtered by a second order high-pass filter at a cutoff frequency of 0.5 Hz, amplified at a gain of 300, and again filtered by a fourth order low-pass filter at a frequency of 50 Hz. The analog filter and gain circuit are designed with a quad operational amplifier (OP497, Analog devices, Norwood, MA, USA). The output signals of the analog circuit are digitalized at a sampling frequency of 1000 Hz using a data acquisition system (NI USB-6229, National Instruments Corporation, Austin, TX, USA). The overall schematic of the data processing system is illustrated in Figure 6.

## 3. Experiment

### 3.1. Instant Donning Experiment

**Figure 7:**
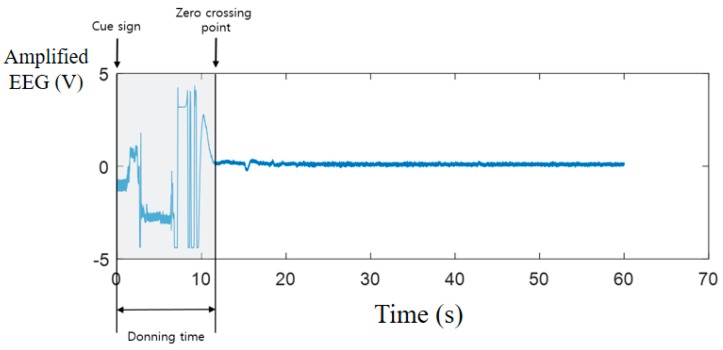
Definition of donning time.

We defined donning time as the time between the cue sign and the first zero crossing point of an acquired signal after donning (Figure 7). The donning time of the channel having the largest value among the eight EEG channels is determined as the donning time of the headset. Four healthy subjects (one female and three male laboratory members, 25 to 34 years old), each with a different hairstyle—specifically, curly, straight, short, and long—participated in the instant-donning experiment with both headsets. This study was approved by the Institutional Review Board of Seoul National University Hospital and all subjects signed informed consent forms to qualify for participation in the study (IRB No. C-1108-088-374). For the posterior-type headset experiment, the subjects were asked to pick up and don the headset after the cue signal. They were then asked to open their eyes for 10 s and close their eyes for 10 s. If there was an alpha rhythm while the subject’s eyes were closed, the EEG was classified as reliable; otherwise, the EEG was a failed measurement after donning the headset. On the other hand, for the around-type headset experiment, an unreliable signal due to unstable contact of an electrode could be observed by spectral analysis. Usually, EEG signals are relatively weak compared with other physiological signals and external electrical signals; therefore, the spectrum of spontaneous EEG signals is typically stable. If the power density of the EEG signal is higher than that of the other signals, then the EEG signal is not classified as reliable. 

### 3.2. Alpha-Rhythm-Based BCI Experiment

The presence of alpha rhythms, which are significant signals in the frequency range of 8–13 Hz during eyes closed, is an indicator of stable and appropriate contact between the electrodes and the surface of the scalp. In this experiment, the presence of alpha rhythms was validated by an alpha-rhythm-based BCI system that is able to control presentation slides by the presence of alpha rhythms. In the experiment, EEG signals from the Oz channel of the posterior-type headset were analyzed and the presence of alpha-rhythm (PoA) determined using the following equation:

(5) $$\mathrm{PoA}=\frac{\sum^{\text{​}}Power_{alpha}}{\sum^{\text{​}}Power_{all_{band}}}=\frac{\sum_{f_{8}}^{f_{13}}P_{EEG}\left(f\right)}{\sum_{f_{2}}^{f_{50}}P_{EEG}\left(f\right)}>Threshold.$$

The four subjects participated in this experiment also. One run consisted of 10 trials and one trial took 3 s. During the experiment, they were asked to close or open their eyes, whichever they wanted to do. If they closed the eyes, the slide would go to the next page; otherwise, it would go to the previous slide if they opened their eyes. The threshold value was empirically determined by the person.

### 3.3. SSVEP-Based BCI Speller

**Figure 8:**
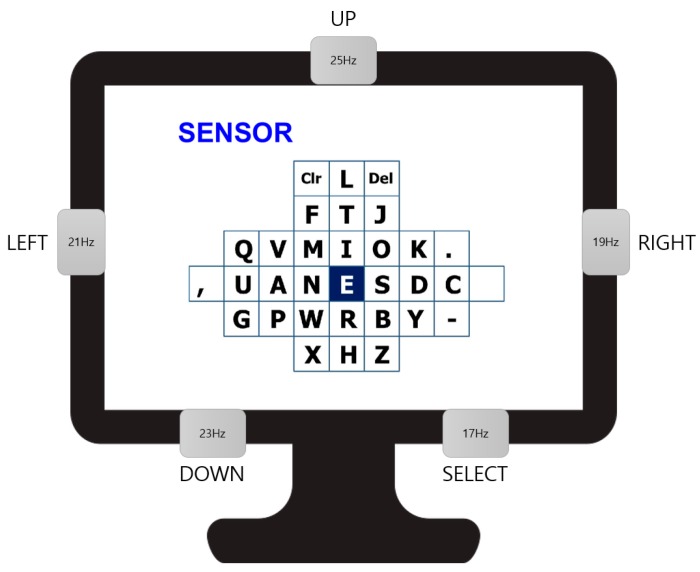
Graphic user interface of Bremen speller and flickering LEDs.

**Figure 9:**
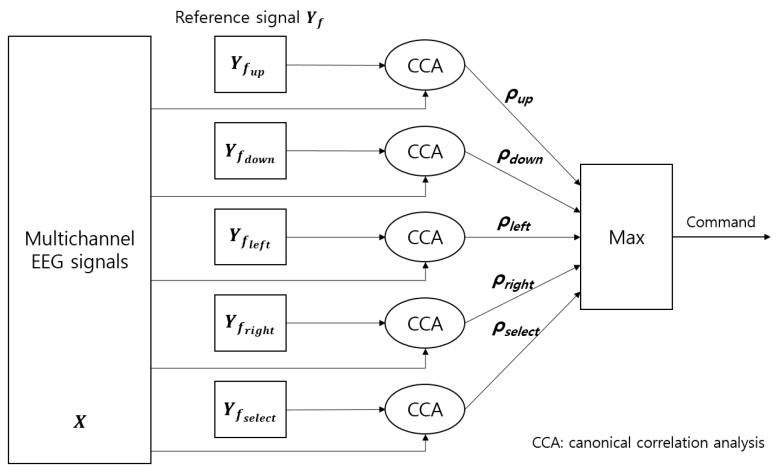
Schematic of the setup used in the SSVEP-based BCI.

A graphical user interface (GUI) was employed for the Bremen BCI speller for SSVEP-based BCI experiments [29,34]. The major feature of this type of speller is that the most often used letters of the English alphabet are arranged in the center to increase the spelling speed, as shown in Figure 8. The five LEDs on the frame of the monitor flickered at 21, 19, 25, 23, and 17 Hz, and corresponded to the commands “LEFT,” “RIGHT,” “UP,” “DOWN,” and “SELECT.” A cursor is located on the letter “E” at the beginning of each trial, and return to the original location is achieved after the “SELECT” command is executed. The canonical correlation analysis (CCA) method was used to determine the desired command [25,35]. This approach is a multivariable statistical method that can be applied to two sets of data that may have some underlying correlation. In this study, one set consisted of the EEG signals from the occipital region, and the other set consisted of periodic sinusoidal signals (Figure 9). 

Each set of the sinusoidal signals consisted of four periodic sinusoidal signals, as defined by Equation (6).

(6) $$Y_{f_{x}}=\left(\begin{array}{c} \sin\left(2\pi f_{x}t\right)\\ \cos\left(2\pi f_{x}t\right)\\ \sin\left(2\pi 2f_{x}t\right)\\ \cos\left(2\pi 2f_{x}t\right) \end{array}\right),\left\{\begin{array}{c} t=\frac{1}{S},\frac{2}{S},\ldots .;\frac{T}{S}\\ x=up,down,left,right,select \end{array}\right\}$$

(7) $$\rho =\underset{i}{\max}\rho_{i},\;i=up,down,left,right,select$$

**Figure 10:**
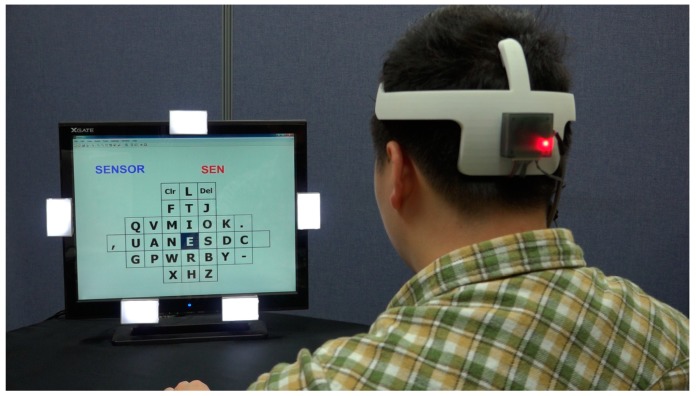
Photograph of the SSVEP-based BCI experiment.

The output command depends on the maximum outcome based on the five combinations elicited by the CCA algorithm. The time window for data acquisition and processing was 6 s. Five healthy subjects (one female and four male laboratory members, 26 to 33 years old) participated in the experiment. Prior to the experiment, they were instructed on how to wear the headset for themselves and how to navigate the cursor to the desired character. The experiment was conducted with each subject seated in a chair approximately 60 cm away from the monitor (Figure 10). All subjects were asked to spell the words “BRAIN,” “ALS,” “NEW,” “SENSOR,” “BCI,” and “SNU.” In the experiment, the posterior headsets were used, and signals from O1 and O2 were processed for SSVEP response detection. To evaluate the overall system, the accuracy, ITR, letters per minute, and efficiency (defined as the minimum number of commands necessary to spell the given word divided by the number of commands issued during the run [36]), were calculated. The ITR is a well-known parameter for BCI system evaluation. This parameter is defined as

(8) $$\mathrm{ITR}=\frac{60}{T}\cdot C_{N}\cdot B_{t},$$

where T denotes the spelling time in seconds, $C_{N}$ is the number of classifications, and $B_{t}$ is derived in accordance with the following:

(9) $$B_{t}=log_{2}N+Plog_{2}P+\left(1-P\right)log_{2}\left[\frac{1-P}{N-1}\right],$$

where *N* denotes the number of targets and *P* is the classification accuracy [29]. It is important to note that the number of targets in our study is not the number of letters in the GUI of the Bremen speller, but rather the number of flickering LEDs (N = 5).

### 3.4. ASSR-Based BCI Paradigm

The experimental paradigm and signal processing of ASSR-based BCI from Kim et al. and Baek et al. [34,37,38] were employed in this experiment. The frequency of the stimulus on the left was a 2.5 kHz pure tone with a modulation frequency of 37 Hz, while the frequency of the stimulus on the right was a 1 kHz pure tone with a modulation frequency of 43 Hz. The around-type EEG headset was used for the experiments for T7 and T8 measurements. Four subjects participated in the experiment. They were visually and verbally instructed to attend to one of the stimuli in random order at the beginning of each trial. The time window for data analyses was 20 s for online experiments. The length of the analysis time affected the overall ITR and accuracy of the BCI system. Therefore, although it is necessary to identify the optimal length of this time window, this task falls outside the scope of this study. The ASSR-based BCI application with instant-donning headsets was evaluated according to the ratio of correct decisions to the total number of trials, specificity, and sensitivity. To calculate the specificity and sensitivity, it was assumed that the right is positive, while the left is negative.

## 4. Results

### 4.1. Instant Donning Experiment

The results of the experiment that quantified the donning time are presented in Table 1. Subject 1 had curly hair and subjects 2, 3, and 4 had straight, short, and long hair, respectively. The average donning time was 10.08 s. Subjects 2 and 3 picked up the headsets faster than subjects 1 and 4 because they had participated in several previous headset experiments. The success and failure of the experiment, i.e., the presence of alpha rhythm, with the posterior-type headset was evaluated. The average success rate was 8.75 out of 10. The average success rate per eight channels with the around-type headset were 7.1/8, 7.8/8, 7.9/8, 7.6/8, respectively. Subject 1, with curly hair, had the lowest success rate. The abbreviation STD signifies the standard deviation.

**Figure 11:**
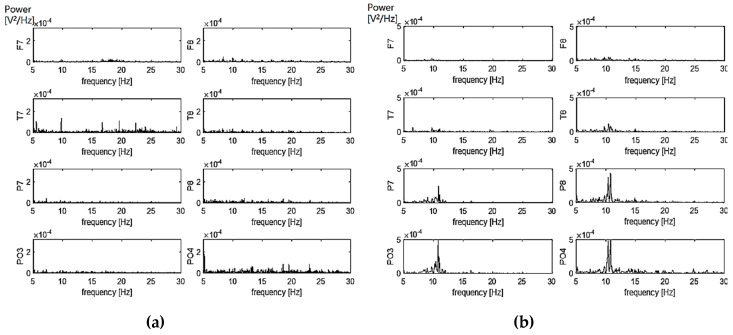
Power spectra of 8 channels of EEG after quick setup: (**a**) eyes-open, (**b**) eyes-closed.

The eight channels of EEG signals were measured for the around-type headset. Spontaneous EEG signals were observed during eyes opened while alpha-rhythms were observed during eyes closed. The power spectrums of each channel are shown in Figure 11a (eye-open) and Figure 11b (eyes-closed).

### 4.2. Alpha-Rhythm-Based BCI Experiment

Table 2 shows the results of the alpha-rhythm-based presentation slides control experiment. As shown in the table, all classification results were corrected, which means that the subjects controlled the presentation slides by opening or closing their eyes and reliable EEG signals were measured immediately after donning of the headsets.

### 4.3. SSVEP-Based BCI Speller

The results of the online experiment are presented in Table 3. The table shows the classification result of each trial and the order in which the given words were completed. The shading commands in the table present wrong classification results that the subjects did not intend. The average of accuracy, ITR, LPM, and EFF were 95.70%, 20.34 b/m 3.75 l/m, and 90.88%, respectively. In comparison with a previous study on SSVEP-based BCI with capacitive electrodes [34], the results obtained in this study are slightly better. It was also observed that the results for the BCI experienced subjects (S1, S2) were better than those for the non-experienced subjects (S3, S4). Considering preparation and donning time, the proposed system has more advantages for BCI application.

### 4.4. ASSR-Based BCI Paradigm

The results of the online ASSR-based experiment are presented in Table 4. The average accuracy, specificity, and sensitivity were 76%, 0.76, and 0.76, respectively. The results of a previous study [34] on ASSR-based BCI system with capacitive electrodes were 72% accuracy, 0.64 specificity, and 0.76 sensitivity. The results obtained in this study are slightly better than that previous study. Considering preparation and donning time, the proposed system has more advantages for BCI application.

## 5. Discussion

We presented two EEG headsets that can be donned quickly and used to immediately measure EEG signals using up to eight channels. Experiments conducted to demonstrate the quick setup for EEG headset with the presence of spontaneous EEG and BCI experiments were conducted. It took approximately 10 s for healthy subjects to don the headsets, including the earclip with the reference and ground electrodes. To the best of our knowledge, such a short preparation and donning time has never been reported previously. Reliable EEG signals were observed after the fast setup. The results of BCI experiments showed the feasibility of the headsets for various BCI applications.

The headsets introduced are designed such that the electrodes can be replaced manually to ensure that they fit the curvature or the shape of the head of the particular subject. However, once the combination of electrodes is set for one subject, it would be difficult to use the headset on another subject; the electrodes would need to be reset for the headset to be used on another subject, which could be time-consuming. To solve this problem, the headsets should be made from material that is more flexible so that the headset can fit the shape of any head more easily after donning it. An experiment with supine patients should be conducted in the future. In this study, only healthy subjects participated in the experiments. This constitutes one of the limitations of the study. Validation experiments with ALS patients who are potential users of the BCI system should be conducted in a separate study.

The proposed headsets measure up to eight channels of EEG signals simultaneously; however, more channels are required for various applications. Therefore, headsets that have more than ten channels should be considered in a future study. In addition, low power consumption, wireless data transmission, and strategies to reduce motion artifacts should be part of a future study on ambulatory monitoring of EEG signals.

Table 5 compares the results obtained in this study with those of several previously published studies. As indicated, the results obtained in this study are similar to (for ASSR) or higher than (for SSVEP) those obtained from previous studies. However, considering the preparation and donning time, the proposed system possesses more advantages for BCI applications.

We can easily search and identify numerous internet video clips on EEG-based BCI applications. However, almost all of these video clips were recorded after the EEG measurement system was set up and prepared to measure the EEG signals. This is obviously because of the prohibitively long time needed for the preparation procedure. By contrast, in this study, the BCI application was conducted with instant-donning EEG headsets. It took approximately 10 s to don the headsets, and reliable EEG signals could be measured immediately. The SSVEP-based and the ASSR-based BCI systems were adopted for the validation experiment. The results obtained with the proposed system are congruent with those obtained in the previous study that used capacitive electrodes [34].

To derive better results in terms of the BCI experiment, signal processing should be considered, such as use of a classification algorithm, and optimization of the time window. However, such work is outside the current scope of this study. 

## 6. Conclusions

To date, various electrode types have been introduced for EEG measurement. However, existing electrodes have inherent drawbacks. With Ag/AgCl electrodes, skin irritation and gel dehydration limit long-term measurement of EEG signals. Consequently, a time-consuming and laborious procedure is required for multichannel measurement with this electrode type. As regards reducing skin irritation, dry-contact electrodes or capacitive coupling electrodes are considered alternative solutions. However, they also have their own drawbacks. Dry-contact electrodes typically have spiky structures to reach to the surface of the scalp passing through the hair. With these structures, however, only the tips contact the surface of the scalp, resulting in high skin-electrode impedance and pain. Capacitive coupling electrodes, on the other hand, do not need direct contact between skin and electrode. However, they are vulnerable to motion artifacts, which results in low signal-to-noise ratios. 

In various EEG-based applications, multichannel EEG measurement is required. However, time-consuming and laborious preparation with assistance limit the measurement to implementation only in a hospital or laboratory. The preparation includes attaching electrodes and gel injection on respective spots on the scalp. This complex procedure for EEG measurement is a bottleneck for practical applications in daily life.

In this study, a new dry EEG electrode structure developed in our previous work was applied to headset. In contrast to existing dry EEG electrodes, it does not have a spiky structure but instead a reversed-curve-arch-shape. The comb-like unique structure increases the skin-electrode contact area on hairy scalps resulting in lower skin-electrode impedance and reduced probability of pain. Two types of quick-setup multichannel EEG headsets were introduced. These two headset types are designed such that all electrodes are able to reach the surface of the scalp while they are being donned, which means that no preparation procedures are required and EEG signals measurement is possible immediately after donning. Validation and BCI experiments with the headsets were conducted to validate the headsets. The results of the validation experiments show that reliable reasoning from EEG signals is possible immediately after donning the headsets without any preparation. Further, the results of SSVEP-based and ASSR-based BCI experiments show that the performance is sufficient for BCI applications, with accuracies of 95.7% and 76.0%, respectively. 

In conclusion, we hope that the developed system will contribute to the acceleration of EEG-based research, such that EEG measurement and applications will become ubiquitous throughout our daily activities.

## Figures and Tables

**Table 1:** Results of donning time quantification experiment (STD: standard deviation).

Trial	Donning Time (s)			
	Subject 1	Subject 2	Subject 3	Subject 4
1	10.11	6.74	11.82	12.85
2	11.70	7.80	7.97	14.68
3	14.19	7.50	8.01	13.46
4	12.66	7.00	6.88	11.42
5	12.90	5.66	6.83	15.22
6	14.95	5.37	10.41	10.95
7	13.18	5.75	7.23	10.43
8	13.98	5.99	7.96	11.53
9	11.98	4.77	9.93	9.75
10	15.98	5.16	9.21	12.83
Mean	13.16	6.17	8.63	12.31
STD	1.70	1.03	1.66	1.80

**Table 2:** Result of alpha-rhythm-based presentation slides control experiment (N: next slide, P: previous slide, spec: specificity, sens: sensitivity).

Subject	Task	Classification Results	Correct/Total	Spec	Sens
1	NPNPNPNPNP	NPNPNPNPNP	10/10	1	1
2	NNNPPNNPNP	NNNPPNNPNP	10/10	1	1
3	NNPNNPNPPN	NNPNNPNPPN	10/10	1	1
4	NNNPPPNNPN	NNNPPPNNPN	10/10	1	1

The time window is 3 s in all tests.

**Table 3:** Classification results of SSVEP-based online BCI experiments (ACC: accuracy, ITR: information transfer rate, LPM: letters per minute, EFF: efficiency, STD: standard deviation).

Subject	Word	Input Results (Dhading: Wrong Result)	ACC (%)	ITR (bit/min)	LPM (letters/min)	EFF (%)
S1	BRAIN	→ ↓ B ↓ R ← ← A ↑ I ← N	100	23.22	4.17	100
	SNU	→ S ← ← → N ← ← ← ← → U	83.33	13.38	2.50	66.67
	BCI	→ ↓ B → → → C ↑ I	100	23.22	3.33	100
	ALS	← ← A ↑ ↑ ↑ L → S	100	23.22	3.33	100
	NEW	← N E ← ↓ W	100	23.22	5.00	100
	SENSOR	→ S E ← N → S ↑ → O ↓ R	100	23.22	5.00	100
S2	BRAIN	→ ↓ B ↓ R ← ← ← → A ↑ I ← N	92.86	18.08	3.57	85.71
	SNU	→ S ← N ← ← ← U	100	23.22	3.75	100
	BCI	→ ↓ B → → → C ↑ I	100	23.22	3.33	100
	ALS	← ← A ← ↑ ↑ → ↑ L → S	90.91	17.01	2.73	81.82
	NEW	← N E ← ↓ W	100	23.22	5.00	100
	SENSOR	→ S E ← N → S ↑ → O ↓ R	100	23.22	5.00	100
S3	BRAIN	→ ↓ B ↓ R ← ← A → ↑ ← I ← N	92.86	18.08	3.57	85.71
	SNU	→ S ← N ← ← ← U	100	23.22	3.75	100
	BCI	→ ↓ B → ← → → ← → → C ↑ I	84.62	13.95	2.31	69.23
	ALS	→ ← ← ← A ↑ ↑ ↑ L → S	90.91	17.01	2.73	81.82
	NEW	← N E ← ↓ W	100	23.22	5.00	100
	SENSOR	→ S E ← N → S ↑ → → ← O ↓ R	92.86	18.08	4.29	85.71
S4	BRAIN	→ ↓ B ↓ R ← ← ← → A ↑ I ← N	92.86	18.08	3.57	85.71
	SNU	→ S ← N ← ← ← U	100	23.22	3.75	100
	BCI	→ ↓ B → → → C ↑ ↑ ↓ I	90.91	17.01	2.73	81.82
	ALS	← ← A ↓ ↑ ↑ ↑ ↑ L → S	90.91	17.01	2.73	81.82
	NEW	← N E ← ↓ W	100	23.22	5.00	100
	SENSOR	→ → ← S → ← E ← N → S ↑ → O ↓ R	93.75	18.60	3.75	75.00
Mean			95.70	20.34	3.75	90.88
STD			5.01	3.39	0.89	11.08

**Table 4:** Classification results of ASSR-based online BCI experiment (Spec: specificity, Sens: sensitivity, shading: wrong classification result, STD: standard deviation)

Subject	Task	Classification Results	Correct/Total	Spec	Sens
S1	RLRLRRRLRL	RRRRLRRRRRRRR	7/10	0.4	1
S2	LRLRLRRLLR	LLLRLLRLLL	7/10	1	0.4
S3	RLLLRRLRLR	RLRLRRLRLR	9/10	0.8	1
S4	RLLRLRLLRR	RLLLLRLLLL	7/10	1	0.4
S5	RRRLLRLLRL	RRRLRRLRRL	8/10	0.6	1
Mean			7.6/10	0.76	0.76
STD			0.09	0.26	0.33

The time window is 3 s in all tests.

**Table 5:** Comparison of the results obtained in this study with those of previous BCI-paradigm studies.

Electrode Type	Preparation Time	Number of EEG Channels	Time Window	Number of Subjects	Accuracy	BCI Paradigm	Study
Gold disk	Several minutes	4	2–20 s	6	84.3%	ASSR	Kim et al. (2011) [38]
Capacitive coupling	Several minutes	4	14 s	5	72.0%	ASSR	Baek et al. (2013) [34]
Wet felt pad	Several minutes	14	6 s	4	83.0%	SSVEP	Liu et al. (2012) [39]
Spike dry	5.7 min	21	†	21	†	†	Halford et al. (2016) [40]
Spike dry	<2 min	3	4 s	10	81.3%	Motor imagery	Lin et al. (2016) [41]
Reverse-curve-arch shaped	~10 s	8	20 s	5	76.0%	ASSR	This study
Reverse-curve-arch shaped	~10 s	8	6 s	4	95.7%	SSVEP	This study

† = not available.
